# Editorial: The Role of the Plasminogen Activating System in Neurobiology

**DOI:** 10.3389/fncel.2016.00222

**Published:** 2016-10-04

**Authors:** Robert L. Medcalf, Daniel A. Lawrence

**Affiliations:** ^1^Molecular Neurotrauma and Haemostasis, Australian Centre for Blood Diseases, Monash UniversityMelbourne, VIC, Australia; ^2^Division of Cardiovascular Medicine, Department of Internal Medicine, University of Michigan Medical SchoolAnn Arbor, MI, USA

**Keywords:** plasminogen, plasminogen activators, blood brain barrier, ischaemic stroke, traumatic brain injury (TBI), neurotoxicity, neuroprotection

The plasminogen activating system has been well-appreciated for its roles in fibrinolysis and metastatic cancer for over 30 years. These observations lead to the clinical development of the key plasminogen activators, namely urokinase (u-PA), and tissue-type plasminogen activator (t-PA) as thrombolytic agents, initially for myocardial infarction in the mid-1980's, and a decade later for use in patients with ischaemic stroke following the approval of tPA (Ninds, 1995). Similarly various attempts were made to modulate cell surface plasminogen activation in an effort to reduce metastatic spread with varying success, although various components of this system have become biomarkers for some malignancies (McMahon and Kwaan, 2015).

While many laboratories continue to work in these classical areas, and with due reason, a growing list of publications dating from the early 1980's revealed that the main components of the plasminogen activating system were expressed in almost all cell types and were regulated by agonists linked to almost all signal transduction pathways identified (Medcalf, 2007). While these reports were consistent with a broadening role of the plasminogen activating system in physiology, other findings also from the early 1980's reported strong expression of components of the plasminogen activating system in the central nervous system (Krystosek and Seeds, 1981; Soreq and Miskin, 1981). While these were largely descriptive studies, and without any clear connection to conventional fibrinolysis or metastatic cancer, speculation arose as to the role of the plasminogen activating system in the CNS (Yepes and Lawrence, 2004), particularly given the fact that the normal brain is devoid of fibrin. A decade or so later, CNS focused reports of activity dependent expression of t-PA in the brain added substantial fuel to notion of a critical role for t-PA in normal brain function, with increases in t-PA gene expression in the CNS correlated with long term potentiation (Qian et al., 1993; Huang et al., 1996); and motor learning (Seeds et al., 1995). Soon after, reports using t-PA deficient mice provided evidence for surprising neurotoxic effects of t-PA where t-PA, via plasmin was shown to be necessary to facilitate glutamate-mediated toxicity *in vivo* (Chen and Strickland, 1997). These reports were published at about the same time that t-PA was approved for therapeutic use in patients with ischemic stroke and raised concerns with the clinical use of t-PA given the fact t-PA administration in ischemic stroke was not risk-free.

It soon became apparent that t-PA was influencing numerous other aspects of brain function including modulation of memory (Huang et al., 1996) and learning (Seeds et al., 2003) and the response to drugs of addiction (Pawlak et al., 2005; Bahi and Dreyer, 2008; Maiya et al., 2009). Another landmark discovery made in the early 2000's reported a potent effect of t-PA at promoting BBB disruption in rodent models of cerebral ischemia (Yepes et al., 2003), an effect that has since been documented in a subset of human stroke patients who receive thrombolysis (Kidwell et al., 2008). This further added to the debate of t-PA as a safe thrombolytic in patients with ischaemic stroke. The enhancing effect of t-PA on BBB permeability not only directed many laboratories to uncover the mechanism behind this (Su et al., 2008; Niego et al., 2012), but also raised interest in other areas of brain pathology where BBB integrity was compromised, namely in traumatic brain injury (TBI, Mori et al., 2001). Initial research into the role of t-PA at influencing outcome following TBI resulted in a number of publications supporting the notion that brain-derived t-PA, as opposed to exogenous t-PA (as in ischemic stroke), was also promoting BBB permeability and subsequent deleterious outcome following TBI (Sashindranath et al., 2012; Su et al.). It soon became apparent that t-PA was indeed a major modulator of BBB permeability (Niego and Medcalf, 2014), even under non-ischemic or traumatic conditions (Fredriksson et al., 2016).

With the realization of these various roles of t-PA in the CNS, questions arose as to how t-PA was implementing these effects and how it was being regulated. t-PA modulating agents i.e., neuroserpin (Lebeurrier et al., 2005), critical signaling systems i.e., tyrosine kinase (Su et al., 2008), and Rho kinase pathways (Niego et al., 2012), and receptors i.e., LRP-1 (Yepes et al., 2003; Samson et al., 2008), and PDGFRα (Fredriksson et al., 2004) in the CNS were later identified by various groups to participate in this new frontier of plasminogen activation biology. Although these findings pushed the field further, controversy also arose. Conflicting reports on how t-PA promoted neurotoxicity (Nicole et al., 2001; Matys and Strickland, 2003; Samson et al., 2008), or its opposite effect (i.e., neuroprotection) via non-proteolytic means (Kim et al., 1999), or proteolytically at low concentrations (Echeverry et al., 2010; Wu et al., 2012) continued to pepper the literature, particularly in recent years (Yepes). The diverse reach of the plasminogen activators in the brain also posed the question as to whether there was a common mechanistic element behind these various, seemingly unrelated events (Fredriksson et al., 2016).

This themed issue of *Frontiers in Cellular Neuroscience* entitled “The role of the plasminogen activating system in Neurobiology” contains 12 contributions from key scientists in this field that includes topics ranging from basic neurobiology, ischaemic stroke, and TBI. Data is presented to implicate t-PA in neurovascular development, how parallel protease systems (i.e., the MMPs) may participate in some aspects of t-PA's effects in the CNS, novel approaches to attenuate t-PA mediated BBB permeability in TBI, and new insights into the biology of the major brain t-PA inhibitor, neuroserpin. We have endeavored to cover areas of controversy, particularly in relation to the purported roles of t-PA at promoting both neurotoxicity and neuroprotection while at the same time include state-of-the-art reviews, including the insights as to how the coagulation and the fibrinolytic systems can modulate the neurovascular unit and how this can in turn have an impact on the immune response.

This themed issue also includes clinical and basic science perspectives which are likely to seed further innovation to future research in this field. At the time of writing this Editorial, these 12 articles have amassed over 15,000 article views and nearly 3000 downloads within ~14 months since publication, providing clear evidence that this particular topic continues to be vibrant, appealing, and important. The relatively recent entry of the plasminogen activator into the field of neurobiology has certainly been an eye-opener. As technology ultimately advances, it is almost certain that the subsequent years will not only uncover novel mechanistic insights into the how the plasminogen activating system functions in the CNS, but it will also uncover important roles for this enzyme system in other key areas of neurobiology.

## Author contributions

All authors listed, have made substantial, direct and intellectual contribution to the work, and approved it for publication.

## Funding

This work was funded in part by funds awarded to RM from the National Health and Medical Research Council of Australia, grant numbers 1045755 and 1045756, and to DL from the National Institutes of Health grant numbers HL055374 and NS079639.

### Conflict of interest statement

The authors declare that the research was conducted in the absence of any commercial or financial relationships that could be construed as a potential conflict of interest.
